# Eiology and prognosis of canalicular laceration repair using canalicular anastomosis combined with bicanalicular stent intubation

**DOI:** 10.1186/s12886-020-01506-w

**Published:** 2020-06-22

**Authors:** Tao Guo, Xiuhong Qin, Hongwei Wang, Yang Lu, Li Xu, Jiali Ji, Caiwen Xiao, Zhenzhen Zhang

**Affiliations:** 1 Department of Ophthalmology, Shanghai Ninth People’s Hospital, Shanghai Jiaotong University School of Medicine, No 639 ZhiZaoJu Road, Shanghai, 200011 China; 2 Department of Ophthalmology, First Affiliated Hospital of Dalian Medical University, Dalian, 116011 Liaoning Province China; 3 Department of Ophthalmology, Jingjiang People’s Hospital, Jingjiang, 214500 Jiangsu Province China

**Keywords:** Canalicular laceration, Etiology, Prognosis, Epiphora

## Abstract

**Background:**

To evaluate the etiology of lacrimal canalicular laceration and explore the possible risk factors influencing prognosis.

**Methods:**

The data of 142 patients (142 eyes) with lacrimal canalicular lacerations who were surgically treated using canalicular anastomosis combined with bicanalicular stent intubation between March 2017 and March 2018 were reviewed. The analyzed data contained demographic information, types of trauma, injury locations, associated additional ocular injuries, and surgical outcomes at follow-up. The main outcome measures were anatomic success rate, functional success rate, and complications of surgery.

**Results:**

The mean patient age was 42.07 years (ranging from 1 to 75 years). Among the 142 patients, 112 (78.87%) were males. Upper and lower canalicular lacerations were found in 14 (9.86%) and 112 (78.87%) patients, respectively. Meanwhile, both upper and lower canalicular lacerations were found in 16 (11.27%) patients. Electric bike accidents comprised the leading cause of injury, accounting for 76 (53.52%) cases. There were 100 (70.42%) patients who had lid lacerations without tarsal plate fracture and 42 (29.58%) patients who had lid lacerations with tarsal plate fractures. The anatomic success rate was 98.59% and the functional success rate was 83.8%. The functional reconstruction failure rates were higher in patients with indirect injuries, lid lacerations with tarsal plate fractures, and those with punctum splitting (*P* < 0.05). Surgical complications were detected in the form of lacrimal punctum ectropion in 3 (2.11%) patients, punctum splitting in 2 (1.41%) patients, and stent extrusion and loss in 2 (1.41%) patients.

**Conclusions:**

Electric bike accidents have become the leading cause of injury instead of motor vehicle accidents because of the changes in the lifestyles of people. Indirect injuries, lid lacerations with tarsal plate fractures, and those with punctum splitting were significantly more likely to lead to poor prognosis, as confirmed by the lower functional success rate of surgery.

## Background

Canalicular laceration, which is commonly regarded as an ocular emergency, is caused by trauma on the eyelids and in periorbital areas. It frequently involves the lower canaliculus and its incidence has been reported in all age groups [1]. Canalicular lacerations are present in approximately 16% of all eyelid lacerations due to ocular trauma [2]. It has been reported that 72% of lower canaliculus lacerations are monocanalicular, whereas bicanalicular lacerations account for 6 to 24% of all canalicular injuries [3]. Based on the mechanisms of damage, Wulc et al. divided canalicular lacerations into direct trauma, such as knife and dog bite injuries, and indirect trauma, such as blunt trauma [4]. It has been reported that the condition of patients with canalicular lacerations due to indirect or diffused injuries can be attributed to factors other than the presence of penetrating injuries [4].

The canaliculus can undergo stenosis, causing lacrimal drainage dysfunction with epiphora, if not appropriately managed [5]. Canalicular anastomosis, combined with bicanalicular or monocanalicular stent intubation, is used for primary canalicular laceration repairs [5]. A variety of materials have been used to stent torn canaliculus clinically [6, 7], such as medical-grade silicone stents (Freda® silicone tube, mini-Monoka®, Masterka®) [8, 9]. The mini-Monoka® is a monocanalicular stent composed of a silicon rod with a bulb and a collar at the proximal end, making it self-retaining [8]. The mini-Monoka® insertion is suitable for conditions such as canalicular laceration involving the external two-thirds of the canaliculus without damaging the canthal ligament. Silicone intubation is most commonly used in surgery because of its advantageous attributes, namely, inert nature, flexibility, and easy availability [6, 7]. Several factors impact the effectiveness of laceration repair, including the extent and location of canalicular lacerations, the intubation materials, the duration of intubation, and the surgical technique [10–12]. The present study was conducted to review the cases of 142 patients with primary canalicular lacerations in the Department of Ophthalmology of Shanghai Ninth People’s Hospital, China. We described the epidemiology and evaluated the etiology and prognosis of primary canalicular laceration repair using canalicular anastomosis combined with bicanalicular stent intubation.

## Methods

### Patients

We retrospectively reviewed the medical records of 142 patients (142 eyes) who had primary canalicular lacerations and required surgical repair within 48 h at the Department of Ophthalmology, Shanghai Ninth People’s Hospital, Shanghai JiaoTong University School of Medicine, Shanghai, China between March 2017 and March 2018. Most of the patients were initially referred to the emergency room, while the others were recruited from clinics. The retrospective study was performed with the approval of the Ethics Committee of Shanghai Ninth People’s Hospital, Shanghai JiaoTong University School of Medicine, China. The informed consent and the commitment to follow-up were signed by all subjects in our study, including the parents or guardians of the study participants who were minors at the time of study. The patients comprised 112 males and 30 females who were 1 to 75 years old (42.07 years on average). Among the 142 patients, 134 received indirect injuries and 8 received direct injuries. The patient demographics, affected canaliculus, number of canaliculus injured, nature of injury, and associated injuries were obtained through patient records. The data on the complications and surgical success rate were also collected for this study, as the post-surgery follow-up visits were recorded at 1.0 week and at 1.0, 2.0, 3.0, and 6.0 months after surgery. The exclusion criteria included lack of adequate follow up (< 3 months), pre-injury epiphora and pyorrhea, additional lacerations involving the lacrimal sac and/or nasolacrimal duct or congenital and/or acquired lacrimal stenosis and/or obstruction.

### Lacrimal system evaluation

We evaluated the lacrimal system before surgery and estimated whether the lacrimal system was involved when the eyelid laceration was situated very close to the medial canthus. Further examination of the lacrimal system was done by irrigation of the lacrimal canaliculi with a 2.0 ml syringe of 0.9% saline solution under topical anesthesia. If the liquid flowed from the wound, a lacrimal probe was used to confirm the position of the distal lacerated end of the lacrimal canaliculus and the distance from the lacrimal punctum and the distal lacerated end was measured.

### Surgical procedure

**Fig. 1:**
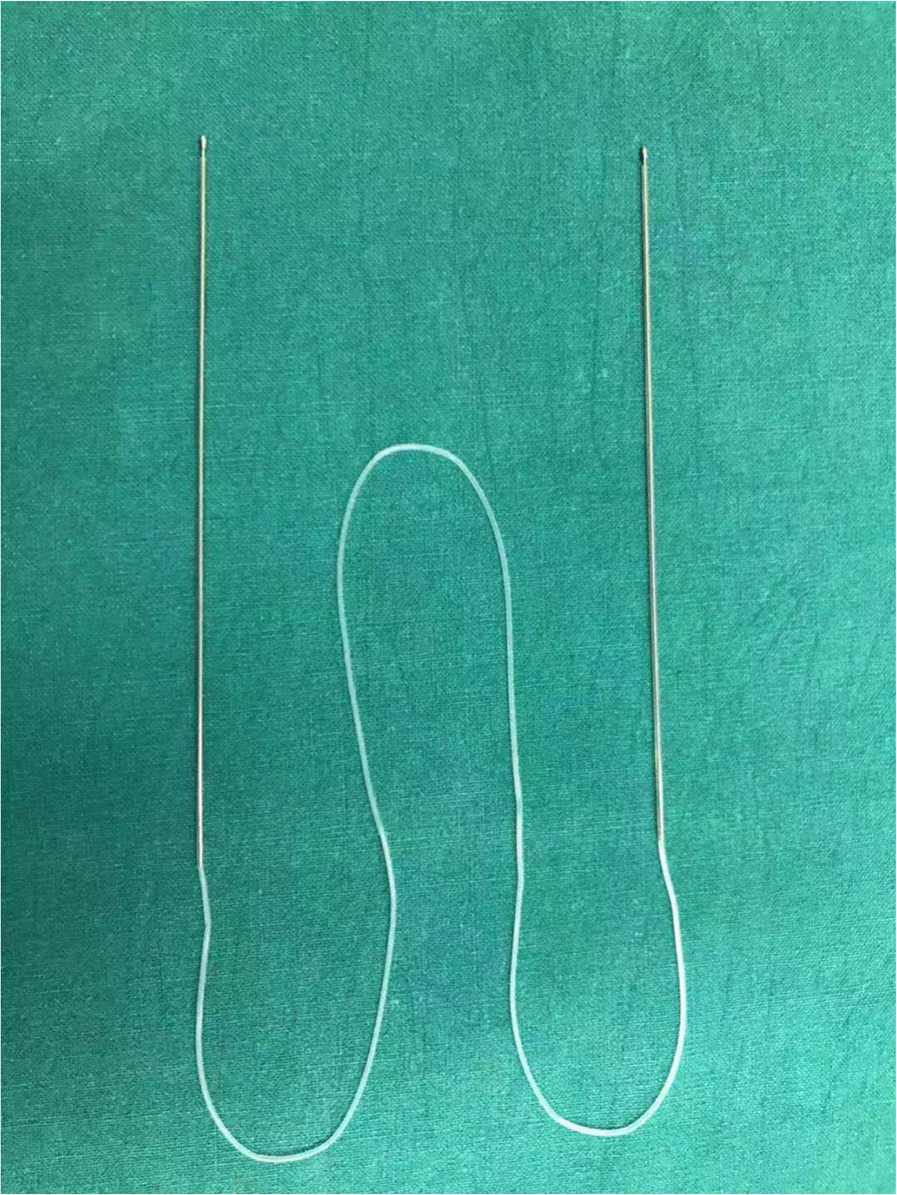
The silicone stent (Shandong Freda Biotechnology Co., Ltd., China)

**Fig. 2:**
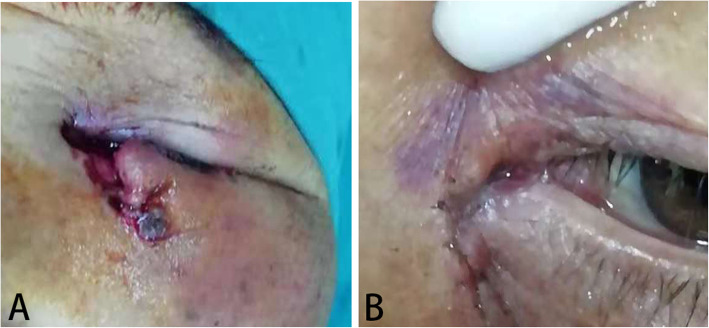
A female patient with lower lacrimal canalicular laceration and full-thickness eyelid laceration of her left eye. **a** Preoperative view of the patient; **b** Postoperative view of the patient by surgery of silicone intubation. Intubation of a bicanalicular silicone stent was seen after surgery

Routine sterilization was conducted and infratrochlear and infraorbital nerve block anesthesia was administered with 2.0 ml of 2% lidocaine and 2.0 ml of 0.75% bupivacaine for adults. General anesthesia was also administered for the pediatric patients. The proximal lacerated end was located with the aid of a surgical microscope (ZEISS, Germany). Then, a punctum dilator was used to enlarge the lacrimal punctum. Bicanalicular silicone tube intubation was done using a 1.0 mm-diameter silicone tube with a probe at both heads (Shandong Freda Biotechnology Co., Ltd., China), as shown in Fig. 1. One head was inserted into the ruptured canaliculus and nasal cavity, while the other end was placed into the upper or lower canaliculus and pulled out from the nasal cavity. The proximal and distal lacerated ends were subsequently anastomosed with 6–0 absorbable suture of polydioxanone (Johnson&Johnson, USA) around the silicone tube. The meticulous re-approximation of the severed canaliculus was performed under an ophthalmic surgical microscope. The two corresponding ends of the silicone tube were tied securely at a proper length. If any globe injury occurred, globe wound repair had to be performed before other management treatments. Repairs of additional eyelid injuries were conducted after the lacrimal intubation. The preoperative and postoperative images of a typical case are provided in Fig. 2. All repairs were performed by the same experienced surgeon.

### Postoperative management

Antibiotics were simultaneously administrated locally and intravenously to prevent infection. Post-surgery follow-up visits were recorded at 1.0 week and at 1.0, 2.0, 3.0, and 6.0 months. The silicone tube was shifted and checked monthly and extubation was performed 3 to 6 months after surgery, followed by lacrimal irrigation. The surgery outcome was defined by lacrimal irrigation and the presence of symptomatic epiphora indoors.

### Statistical analysis

Data were presented as mean + SD or *n* patients. The SPSS 22.0 software was used for statistical analysis. The clinical prognoses and surgical outcomes of the canalicular lacerations were compared using Chi Square test. Kaplan–Meier analysis and Cox proportional hazards regression analysis were used to determine the risk factors influencing the prognosis of canalicular laceration. All *P* values were considered statistically significant when the values were < 0.05.

## Results

In our study, 112 (78.87%) of the patients were males and 30 (21.13%) were females. The average age was 42.07 years (ranging from 1 to 79 years). A total of 126 (88.7%) patients had one canaliculus involved, 16 (11.3%) had 2 canaliculi involved, and no patient had 3 or 4 canaliculi involved. Upper and lower canalicular lacerations were found in 14 (9.86%) and 112 patients (78.87%), respectively, while both canalicular lacerations were found in 16 patients (11.27%). The mean time interval between injury and surgery was 14.42 + 0.36 h (from 3 to 48 h). The mean time of canalicular stent removal was 4.5+ 0.54 months and the mean follow-up period was 6.94 + 0.51 months (Table 1).

**Table 1:** Clinical characteristics of patients undergoing Canalicular laceration repair

***Variable***	Patient numbers	Proportion
Total patients	142	
Mean age	42.07(from1–79 year)	
Males	112	78.87%
Females	30	21.13%
Eye involved	142	
Right	91	64.08%
Left	51	35.92%
Canaliculus involved		
Upper	14	9.86%
Lower	112	78.87%
Both	16	11.27%
Mean time between injury and repair	14.42 + 0.36(from 3 to 48 h)	
Mean time of stent removal	4.5 + 0.54(from 3 to 6 months)	
Mean follow-up period	6.94 + 0.51(from 6 to 9 months)	
Indirect injuries	134	
Electric bike accidents	76	53.52%
Blunt injuries	32	22.54%
Car accidents	10	7.04%
Falls	12	8.45%
Fights	4	2.82%
Direct injuries	8	
Sharp objects	6	4.22%
Dog bites	2	1.41%
Additional injuries		
Lid laceration without tarsal plate fracture	100	70.42%
Lid laceration with tarsal plate fracture	42	29.58%
Lid laceration with lacrimal punctum splitting	6	4.23%
Extraocular muscle injuries	14	9.86%
Head trauma	10	7.04%
Ptosis	7	4.93%
Globe rupture	6	4.23%
Optic neuropathy	2	1.41%
Vitreous and/or retinal detachment	2	1.41%
Surgery complication		
Lacrimal punctum ectropion after surgery	3	2.11%
Lacrimal punctum splitting after surgery	2	1.41%
False path	0	0%
Stent extrusion and loss	2	1.41%

Data presented as mean + SD (range) or *n*(%)

The types of trauma that caused the canalicular lacerations are shown in Table 1. Among the patients, indirect canalicular injuries were detected in 134 (94.4%), which were remarkably more frequent than direct injuries, which were detected in only 8 (5.6%) patients. Electric bike accidents comprised the leading cause of injury, accounting for 76 (53.52%) patients. The other mechanisms of injury were blunt injuries for 32 (22.54%) patients, car accidents for 10 (7.04%) patients, fights for 4 (2.82%) patients, falls for 12 (8.45%) patients, sharp objects for 6 (4.22%) patients, and dog bites for 2 (1.41%) patients, as shown in Table 1.

Other additional injuries associated with trauma that afflicted the patients are also presented in Table 1. There were 100 (70.42%) patients who had lid lacerations without a tarsal plate fracture and 42 (29.58%) patients with tarsal plate fractures. Canalicular laceration combined with globe rupture occurred in 6 (4.23%) patients in terms of additional injuries. Some patients may have experienced two or more additional injuries at the same time. Other injuries associated with the trauma are as follows: 14 (9.86%) extraocular muscle injuries, 10 (7.04%) head trauma, 7 (4.93%) ptosis, 2 (1.41%) optic neuropathies, and 2 (1.41%) vitreous and/or retinal detachments (Table 1).

All of the canalicular lacerations were repaired during this study. The mean time of canalicular stent removal was 4.5 + 0.54 months. During the follow-up visits, there were two patients with stent extrusion and loss because of a loose knot and because the patients had pulled the suture out. No patient suffered from infection of the lacrimal canaliculus during the visits.

The surgery outcomes of the canalicular lacerations are presented in Table 2. After stent removal, the patients underwent irrigation of the lacrimal canaliculus. They were asked about epiphora during the follow-up. All 142 patients exhibited anatomic success, notwithstanding the two patients with stent extrusion and loss. Among the patients who demonstrated anatomic success, 119 (83.8%) were cases of functional success, claiming no epiphora. As shown in Table 2, among the upper, lower, and both canalicular laceration repair surgeries, there was no significant difference between the anatomic success and functional success rates (*P* > 0.05; *P* > 0.05). The data also showed no significant difference in the anatomic success rate between indirect and direct injuries.

**Table 2:** Outcomes of canaliculus anastomosis and bicanalicular stent intubation

Parameters	Patients	Anatomic success	Functional success
Canaliculus anastomosis and bicanalicular stent intubation	142	140 (98.59%)	119 (83.80%)
Upper	13	12 (92.31%)	10 (83.33(%)
Lower	113	111 (98.23%)	96 (86.49%)
Upper and lower	16	15 (93.75%)	13 (86.67%)
*P*		> 0.05	> 0.05
Indirect injuries	134	132 (98.51%)	101 (75.37%)
Direct injuries	8	8 (100%)	7 (87.5%)
*P*		> 0.05	< 0.01
Additional injuries			
Lid laceration without tarsal plate fracture	100	99 (99%)	78 (78%)
Lid laceration with tarsal plate fracture	42	41 (97.62%)	30 (71.43%)
*P*		> 0.05	< 0.01
Lid laceration without lacrimal punctum splittting	136	134 (98.53%)	105 (77.21%)
Lid laceration with lacrimal punctum splittting	6	6 (100%)	3 (50%)
*P*		> 0.05	< 0.01

**Fig. 3:**
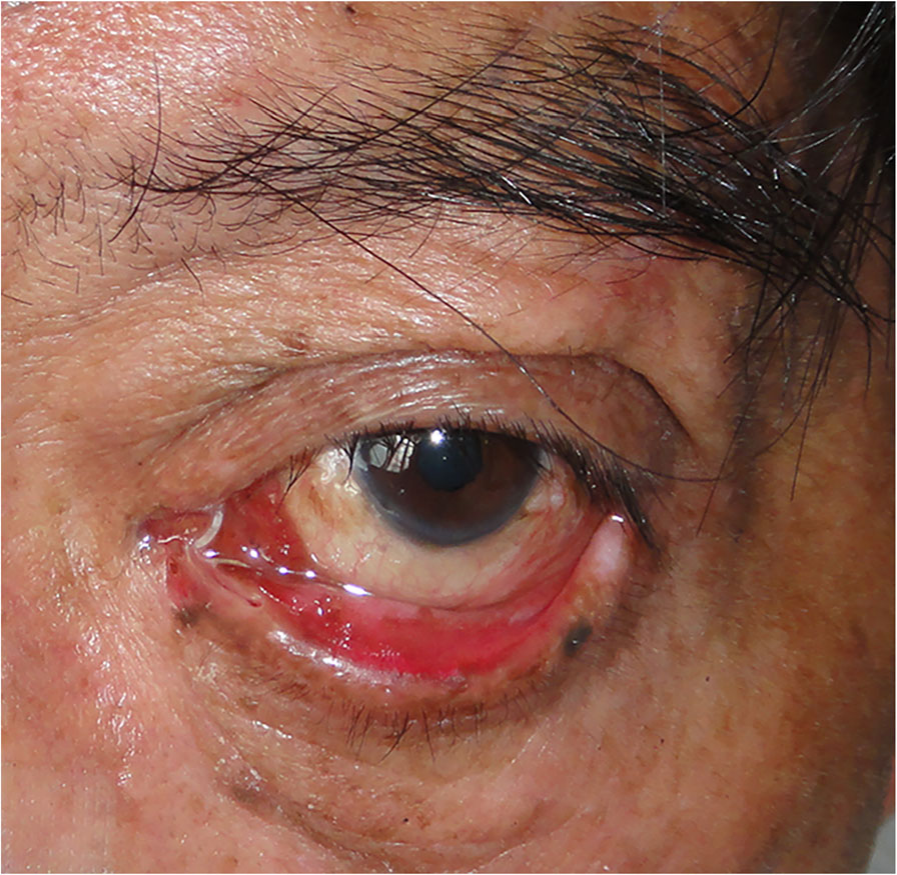
The figure of complication with lacrimal punctum ectropion and splitting

**Fig. 4:**
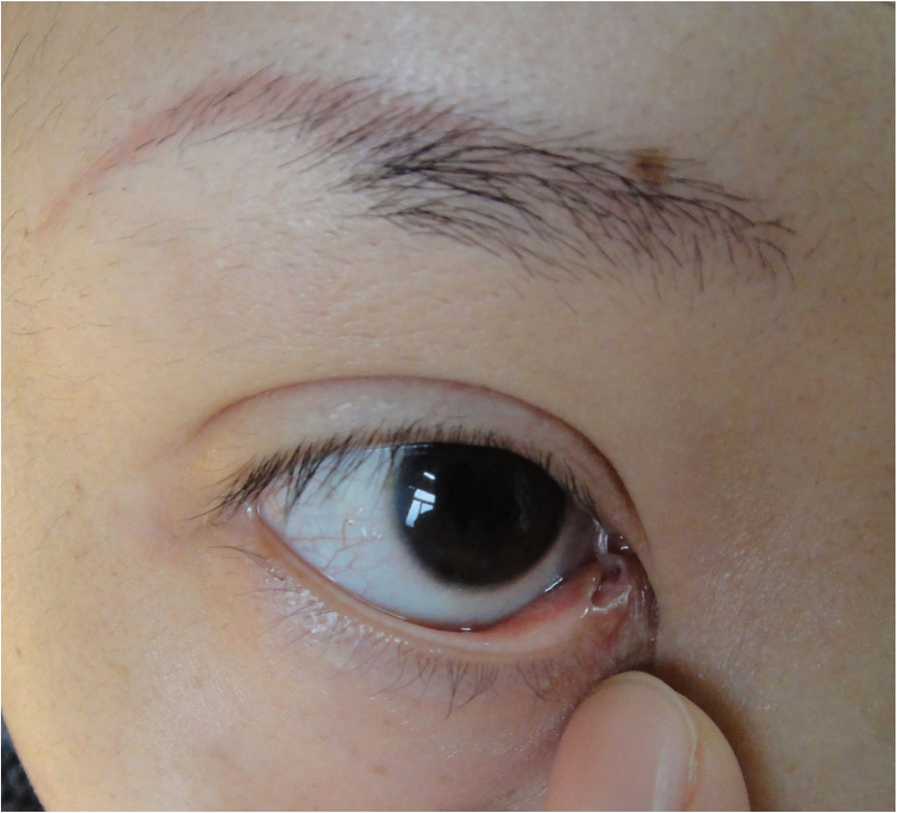
The figure of complication with lacrimal punctum splitting

As shown in Table 2, the functional success rate was significantly lower in indirect injuries than direct injuries (*P* < 0.01). Between the canalicular laceration with and without tarsal plate fracture, there was no significant difference in anatomic success rate. However, surgery had a higher functional success rate in the canalicular lacerations without tarsal plate fracture than in those with tarsal plate fractures (*P* < 0.01). Between the canalicular laceration with and without punctum splitting, no significant difference was shown in the anatomic success rate. However, surgery had a higher functional success rate in the canalicular lacerations without punctum splitting than in those with punctum splitting (*P* < 0.01). Regarding the surgery complications, we only found 3 (2.11%) patients with lacrimal punctum ectropion (Fig. 3) and 2 (1.41%) patients with punctum splitting (Fig. 4). No patient had a false path.

The results of the Kaplan–Meier analysis for treatment success rate are shown in Table 3. The Cox proportional hazards regression analysis of prognostic factors in canaliculus laceration repair surgery are also presented. Notably, canalicular lacerations with indirect injury, tarsal plate fracture, and punctum splitting were significantly more likely to have poor prognosis (*P* = 0.017, 0.036, and 0.045).

**Table 3:** Cox proportional hazards regression analysia of risk factors for the canalicular lacerations

Risk factors	Hazard ratio(95% CI)	Statistical signifiance
Canaliculus involved	0.973 (0.901, 1.046)	NS
Indirect injuries	1.062 (1.005, 1.097)	*P* = 0.017
Lid laceration with tarsal plate fracture	0.641 (0.157, 0.965)	*P* = 0.036
Lid laceration with lacrimal punctum splitting	32.783 (1.091, 2475.563)	*P* = 0.045
Globe rupture	1.371 (0.255, 6.478)	NS

Data are based on 142 Chinese patients with lacrimal laceration

*CI* Confidence interval

*NS* Not statistically significant(*P* ≥ 0.05)

## Discussion

Canalicular laceration commonly accompanies facial trauma and requires early intervention (within 48 h) in the ophthalmology department to restore anatomy and function [4]. Canalicular lacerations are pervasive among males, accounting for about 78.87% in our study, similar to the results of Naik et al., who reported 86% male cases [8]. In this study, patients with lower canalicular laceration involvement were predominant (78.87%). Liang et al. reported that 82.9% had lower canalicular lacerations, 11.4% had upper canalicular lacerations, and 5.7% had bicanalicular lacerations in their studies [13]. Our data corresponded with the findings of the above studies. Our mean time between injury and repair is 14.42+ 0.36 h (from 3 to 48 h). Some authors posit that early treatment (9–32 h) is the key to success in canalicular repair [8, 14]. Tint et al. showed poor outcomes in 6 out of 40 patients with delayed repair (2–3 days) [14]. By contrast, Chatterjee et al. reported five patients who presented between 2 to 4 days since their injuries but still had successful outcomes after surgery [15].

Although the epidemiology of canalicular laceration had been published in some reports, the types of traumas that cause these injuries have changed due to the altered lifestyles of people. In our study, electric bike accidents comprised the leading cause of injury, accounting for 76 (53.52%) patients, instead of motor vehicle accidents (35.81%), as was the case in the past [16]. Electric bikes have replaced motor vehicles due to their advantageous attributes, such as being inexpensive and environment friendly. With the increase in the number of electric bikes, the related rate of accidents is also increasing. We also found that patients with indirect canalicular injuries were remarkably more predominant than those with direct injuries, similar to the results derived by Wulc et al. [4].

Our study showed that the rate of patients who had lid lacerations with tarsal plate fractures was 29.58%. As there are no previous data on the incidence of tarsal plate fracture during injury, we concluded that lid lacerations without tarsal plate fracture are more frequent than those with tarsal plate fractures. In our study, globe rupture occurred in 6 (4.23%) patients. Herzum et al. reported a 20 to 44% incidence rate for globe injury in association with eyelid injuries [2]. These results are quite different from our own. Lee et al. later reported that traumatic hyphema and subconjunctival hemorrhage represented the most frequent associated ocular injuries instead of globe injury [17].

It is believed that the key to a successful surgical repair of canalicular laceration is to find the proximal lacerated end quickly and precisely [18]. Several methods and techniques to identify the proximal lacerated end of the canaliculus, such as pigtail probe [19], upper canalicular probing, and bubble or colored opaque solution injection, have been presented in previous studies [17, 18, 20, 21]. Silicone intubation is the strategy that is most commonly used in surgery because of its capability of restoring a normal anatomical pathway to avoid a false path [5]. With double-passage canalicular intubation, circular stents using silicone tubes provide good stabilization, preserve the natural location of the medial canthus, and maintain the physiological anatomical reposition of the superior and inferior punctum, thereby preventing ectropion and laceration of the lower eyelid and inferior punctum. It also offers excellent tear drainage [5–7]. However, the disadvantages of double-passage canalicular intubation include symptoms of irritation and additional secretion [22]. All canalicular lacerations in our cases were repaired through double-passage canalicular intubation with successful anastomosis.

Among these patients, 140 (98.59%) had anatomic success and 119 (83.8%) had functional success. Kersten et al. described an alternative surgical approach for the repair of canalicular laceration using silicone tube intubation with a success rate of 96% based on lack of symptomatic epiphora [20]. Liang et al. reported that 91.18% of their patients experienced complete success with total disappearance of epiphora and 8.82% eyes achieved partial success after tube removal [13]. The results we obtained are similar to the literature reported above. Our results showed that certain factors, namely, indirect injuries, lid laceration with tarsal plate fracture, and those with lacrimal punctum splitting, led to lower functional success rate of surgery and were the risk factors for canalicular laceration repair surgery. The reason may be the severe scarring surrounding the canaliculus due to the tarsal plate fracture and lacrimal punctum splitting.

## Conclusions

The causes of canalicular lacerations have changed because of the altered lifestyles of people. Our studies showed that certain factors, namely, indirect injuries, lid lacerations with tarsal plate fractures, and those with punctum splitting, led to lower functional success rate of surgery and were the risk factors for functional reconstruction after repair surgery. A drawback of this study is its retrospective, noncomparative nature. A large-scale study of a comparative nature is needed in the future. The results of the present study will provide some suggestions for the prognosis of surgical treatment for canalicular laceration.

## Data Availability

The datasets of the current study are available upon request from the co-correspondence author Jiali Ji and CaiWen Xiao.
